# Editorial

**Published:** 1870-01

**Authors:** 


### Editorial.

A NEW-YEAR AND A NEW VOLUME.—The present number commences the 11th volume of the EXAMINER, and with it we wish all our readers a "HAPPY NEW YEAR" and abundant success in their daily work of alleviating human suffering, and of prolonging human life. If the contents of this journal, as it visits them from month to month, shall afford them any aid in their noble work, one of the leading objects of its publication will have been attained.

We shall not follow the example of some of our cotemporaries and boast of past success, or make extravagant promises for the future. The EXAMINER is neither the organ of a school nor the advertising medium of a publishing or bookselling house, but is simply the individual property of its Editor, and is published solely for the promotion of the educational, scientific, and practical interests of the profession. We shall make it as useful to our readers as we possibly can, being fully satisfied with the liberal patronage it has hitherto received.

Medical College Education.—In another place will be found a notice from the Committee appointed by the Medical College Convention, of 1866, calling for a convention of delegates, from all the regular medical colleges in the United States, in the City of Washington, on Friday preceeding the next regular meeting of the American Medical Association. The same notice has been sent to all the medical journals in the country, and we hope those controlling the medical press will lend their aid in bringing together so full a representation from the colleges, that whatever measures are agreed upon, can be successfully carried into practical operation. We know of no candid and enlightened medical teacher who does not acknowledge that there are very important defects in the present system of medical college instruction in this country. This much, indeed, seems to be universally acknowledged by the profession. The time appointed for the Convention will allow of three working days before the assembling of the American Medical Association, which is none too long for a fair consideration of the several important questions that will require consideration. Let us not only have a full convention, but let the delegates come together in a spirit of liberality and with a positive desire to make such changes in the prevalent system of medical college instruction as the interests, the honor, and the usefulness of the profession require, and there will be no difficulty in bringing about important practical results.

---

Diploma Venders.—We have been kindly furnished with two or three of the following cards, by friends in different parts of the country:—

"The Milwaukee Medical and Surgical Institute (chartered by the Legislature of Wisconsin), office, corner of East Water and Mason Streets. Collegiate agency.

"This agency has been established for the purpose of giving such information as is necessary before taking any of the learned degrees in arts, science, law, and medicine, and represents some of the best universities in America and Europe.

"By or through its recommendation, the degrees of A.M.

(Master of Arts), A.B. (Bachelor of Arts), L.L.D. (Doctor of Laws), M.D. (Doctor of Medicine), D.D.S. (Doctor of Dental Surgery), etc., may be legitimately conferred.

"For particulars address the Principal, T. Williams, M.D., Milwaukee, Wis.

"Many States have passed laws prohibiting physicians, who are not graduates, from practising medicine, collecting fees, or giving evidence in Court on medical matters. All the other States will soon pass similar laws. Similar qualifications are required of surgeons of the U. S. Army. In view of the agitation of this question, and the facts that no physician without a diploma can be recognized as a *bona fide* member of the profession, and that it is a passport to good fellowship with his professional brethren and the confidence of the community, every practising physician should be able to produce this badge of his profession."

Will some of our medical friends in Milwaukee give us reliable information in regard to this matter? Is there any such chartered institution in Milwaukee? Is there any such individual as T. Williams, M.D.? If so, who and what kind of man is he? Let this imposition be fully investigated and exposed.

### FOREIGN ITEMS.

*Gazette, September 25th.*—The annual report of Dr. Aubert Roche, on the sanitâry and medical condition of the works and establishments of the Isthmus of Suez, states, that the population of the isthmus, which, in 1859, consisted of 180 persons—25 Europeans and 155 natives—numbered last year 34,258 individuals—16,010 Europeans and 18,248 natives—and that it is at present actually estimated at 42,400, or 22,843 Europeans and 19,559 natives.

The mortality, except in cholera years, has maintained an average of 1 in 100, while in France the rate is 2.40 per 100.

In closing this report, the Dr. says: "During the ten years of our service here, the public health has been constantly improving, the diseases and the mortality have diminished. But at what a painful price have we arrived at this result!

"This year, again, four of our physicians have succumbed, three on the field of battle, and the fourth, Dr. Pappatheodoro, after eight years of service has died in his own country. Of the eleven physicians who were the first participants in our enterprise, there does not remain more than five. The sanitary service has lost more than half of its effective force in chiefs of service."

*Gazette, September 25th.*—By the last mail from India, we learn that some cases of cholera have occurred at Madras. The epidemic seems to have been imparted to travellers coming from the district of Bellary. The sanitary and municipal authorities of Madras are exerting the most energetic measures to prevent the spread of the disease.

*Gazette, September 30th.*—The mortality report, for Paris, for the week ending September 25th, gives deaths from variola 15, scarlatina 5, rubeola 4, typhoid fever 32, typhus 0, erysipelas 6, bronchitis 24, pneumonia 37, diarrhœa 28, dyssentery 6, cholera 9, angina catarralis 4, croup 1, puerperal affections 3, other causes 646—total 820.

The report, for London, for the week ending September 18th, gives deaths from variola 6, scarlatina 178, rubeola 21, typhoid fever 33, typhus 6, erysipelas 4, bronchitis 63, pneumonia 45, diarrhœa 139, dyssentery 2, cholera 5, angina catarralis 13, puerperal affections 10, other causes 874—total 1400.

---

### MEDICAL COLLEGE CONVENTION.

---

To the Trustees and Faculties of the Medical Colleges in the United States:

The undersigned Committee, in accordance with the instructions of the Convention of Delegates from Medical Colleges, held in Cincinnati, in May 1866, respectfully and earnestly invite you to send delegates to a convention, to be held in the City of Washington, on *Friday* preceding the *first* Tuesday in May, 1870, for the purpose of considering all subjects connected with Medical college education, and procuring the coöperation of the Schools in carrying out a uniform system of instruction. It is very desirable that every regular medical college in this country should be represented in the Convention.

CHICAGO, ILL., *Dec. 22, 1869.*

N. S. DAVIS,

S. D. GROSS,

GEO. C. BLACKMAN,

F. DONALDSON,

} *Committee.*

---

FOUR PRIZES.—The Editors of the *American Journal of Obstetrics and Diseases of Women and Children* offer the following prizes for the best essays on the subjoined subjects:—

1. Fifty dollars (in gold) for the best essay on "Catarrh of the Uterus, its Etiology and Treatment."

2. One hundred dollars (in gold) for the best essay on "The Morbid Anatomy of the Placenta."

3. Fifty dollars (in gold) for the best essay on "Electricity in the Treatment of the Diseases of Infants and Children."

4. One hundred dollars (in gold) for the best easay on "Congenital Deformities and Diseases Depending on Maladies of the Uterus or Membranes."—*Buffalo Med. and Surg. Journal.*

---

MODERN HOMŒOPATHY.—From the *London Monthly Homœopathic Review* for June, 1868, we learn that Lord Ebury gave vent to a feeling of regret that the report of the London Homœopathic Hospital did not contain evidence of a greater development of the objects of the institution. The number of patients was not very large, and the clinical lectures had been given up, "owing to the attendance being so scanty as greatly to discourage the lecturers." In the London Homœopathic Hospital, in which the cases are not of so serious a character as one is accustomed to see under treatment in hospital, black wash is applied to syphilitic sores, and ½-grain doses of mercury given internally. Cases of glandular enlargement in the neck were treated with tincture of iodine painted on externally. For cases of continued sleeplessness 1.48 of a grain of acetate of morphia was given every fifteen minutes. The Hospital physicians repudiated, one and all, the fallacies and absurdities of Hahnemann about infinitesimal doses and dilutions.—*N. Y. Medical Record.*

MORTALITY FOR THE MONTH OF NOVEMBER, 1869:—

| | |
|---|---|
| Accident, concussion of brain | 1 |
| " drowned | 3 |
| " by fall | 1 |
| " fall of iron column | 1 |
| " in plaining mill | 1 |
| " injury, spine | 1 |
| " fracture, " | 1 |
| " railroad | 3 |
| " scalded | 3 |
| " smothered in bed | 1 |
| " shot, revolver | 1 |
| Abscess, perineal | 1 |
| Arosophagus, stricture | 1 |
| Apoplexy | 4 |
| Ascitis and anasarca | 1 |
| Asthma | 3 |
| Births, premature | 12 |
| " still | 51 |
| " tedious | 1 |
| Bowels obstruction | 1 |
| Brain congestion | 2 |
| " " delirium tremens | 1 |
| " disease of | 3 |
| " inflammation of | 5 |
| " " typhoid fever | 1 |
| " softening of | 1 |
| Bronchitis | 3 |
| " and dropsy | 1 |
| " chronic | 3 |
| Carbuncle on back of head | 1 |
| Cerebritis | 1 |
| Cholera infantum | 2 |
| Convulsions | 40 |
| Consumption | 48 |
| " pneumonia | 1 |
| Coxitis and pyæmia | 1 |
| Croup | 14 |
| " diphtheretic | 1 |
| " membranous | 3 |
| Cyanosis | 2 |
| Debility | 5 |
| Delirium tremens | 3 |
| Diabetis melitis | 1 |
| Diarrhœa | 3 |
| " chronic | 2 |
| Diphtheria | 13 |
| Dropsy | 2 |
| Dysentery | 5 |
| " chronic | 1 |
| Encephalitis | 2 |
| Endo-pericarditis | 1 |
| Entero-colitis | 1 |
| Enteritis | 5 |
| " and convulsions | 1 |
| Exposure | 1 |
| Erysipelas | 2 |
| Face, cancer of | 1 |
| Fever, congestive | 1 |
| " intermittent | 1 |
| " puerperal | 4 |
| " remittent | 2 |
| " scarlet | 64 |
| " " bronchitis | 1 |
| " " diphtheria | 1 |
| " " dropsy | 1 |
| " " nephritis | 1 |
| " typhoid | 29 |
| Gangrene and anasarca | 1 |
| Gastritis | 3 |
| Hæmatemesis | 1 |
| Heart, disease of | 8 |
| " neuralgia of | 1 |
| " organic disease of | 1 |
| " valvular disease | 2 |
| " fatty degeneration of | 1 |
| Hydrocephalus | 6 |
| " acute | 2 |
| Inanition | 7 |
| " and miscarriage | 1 |
| Intemperance | 2 |
| Jaundice | 1 |
| Kidneys, disease of | 1 |
| Laryngitis | 1 |
| Lead-poisoning | 1 |
| Liver, congestion of | 2 |
| " obscure disease | 1 |
| " fatty degenerat'n | 1 |
| Lungs, congestion of | 4 |
| Measles | 1 |
| Manslaughter | 1 |
| Metro-peritonitis | 1 |
| Meningitis | 4 |
| " cerebro-spinal, | 1 |
| " tubercular | 6 |
| Metritis | 3 |
| Old age | 14 |
| Œsophagus, canker of | 1 |
| Œdema pulmonum | 1 |
| Paralysis | 5 |
| Pericarditis | 1 |
| " and rheumatism | 1 |
| Peritonitis | 4 |
| " metritis | 1 |
| " stricture of intestines | 1 |
| " puerperal | 3 |
| Phrenitis | 1 |
| Pneumonia | 29 |
| " typhoid | 3 |
| " pleurisy | 1 |
| Rheumatism | 1 |
| " inflammatory | 1 |
| Scrofula | 2 |
| Suicide poisoning | 2 |
| Shooting | 1 |
| Syphilis, hereditary | 3 |
| Tabes mesenterica, | 9 |
| Teething | 7 |
| " and bronchitis | 1 |
| " encephalitis | 1 |
| " pneumonia | 1 |
| Tetanus from wound to foot | 1 |
| Thrush | 1 |
| Trismus | 1 |
| Urethra, rupture of, extravasation of urine, result of | 1 |
| Vitality deficient | 1 |
| Whooping-cough | 5 |
| Unknown | 3 |
| Total | 489 |

AGES.

| | |
|---|---|
| Under 1 | 119 |
| 1 to 3 | 81 |
| 3 to 5 | 28 |
| 5 to 10 | 40 |
| 10 to 20 | 23 |
| 20 to 30 | 48 |
| 30 to 40 | 40 |
| 40 to 50 | 38 |
| 50 to 60 | 26 |
| 60 to 70 | 30 |
| 70 to 80 | 9 |
| 80 to 90 | 4 |
| 90 to 100 | 3 |
| Total, | 489 |

| | | | | | |
|---|---|---|---|---|---|
| Males, | 287 | Females, | 202 | Total, | 489 |
| Single, | 355 | Married | 134 | Total, | 489 |
| White, | 481 | Colored, | 8 | Total, | 489 |

COMPARISON.

| | | |
|---|---|---|
| Deaths in Nov., 1869, 489 | Deaths in Nov., 1868, 402 | Increase, 87 |
| Deaths in Oct., 1869, 601 | Decrease, 112 | |

NATIVITY.

| | | | | | |
|---|---|---|---|---|---|
| Bohemia, | 3 | England, | 10 | Sweden, | 9 |
| Canada, | 7 | France, | 3 | Scotland, | 2 |
| Chicago, Native, | 73 | Germany, | 60 | Unknown, | 8 |
| Chicago, Foreign, | 146 | Ireland, | 71 | | |
| U. S., other parts, | 88 | Norway, | 6 | Total, | 489 |
| Denmark, | 2 | Prince Ed'd's Island | 1 | | |

MORTALITY BY WARDS FOR THE MONTH.

| Wards. | Mortality. | Pop. in 1868. | One death in |
|---|---|---|---|
| 1 | 6 | 9,094 | 1515¾ |
| 2 | 23 | 13,074 | 568½ |
| 3 | 26 | 15,076 | 579 4-5 |
| 4 | 20 | 17,796 | 889¾ |
| 5 | 37 | 16,033 | 433⅓ |
| 6 | 24 | 13,083 | 545 |
| 7 | 46 | 25,492 | 554⅙ |
| 8 | 35 | 15,813 | 451 4-5 |
| 9 | 29 | 19,297 | 665½ |
| 10 | 13 | 12,925 | 994¼ |
| 11 | 21 | 14,340 | 683 |
| 12 | 56 | 17,485 | 312¼ |
| 13 | 18 | 11,164 | 620¼ |
| 14 | 26 | 14,839 | 570 4-5 |
| 15 | 30 | 21,078 | 702½ |
| 16 | 20 | 15,465 | 773¼ |

| Mortality. | |
|---|---|
| Accidents, | 17 |
| County Hospital, | 12 |
| Home for Friendless, | 1 |
| Immigrants, | 3 |
| Manslaughter, | 1 |
| Mercy Hospital, | 8 |
| Protestant Orphan Asylum, | 6 |
| St. Joseph Orphan Asylum, | 3 |
| St. Luke's Hospital, | 1 |
| Hospital Alexian Brothers, | 1 |
| Madison Street Police Station, | 2 |
| Twenty-Second St. " " | 1 |
| Suicide, | 3 |
| Total, | 489 |

---

On the Medicinal Use of Phosphorus and its Compounds.—We take the following extracts from an article, with the above caption, by John C. Thorowgood, M.D., published in the *Practitioner*, for July, 1869:—

"Since the discovery and isolation of the element phosphorus by Brandt, of Hamburgh, in 1669, it has become the practice with physicians, in this and other countries, occasionally to prescribe this substance, as a remedy in cases where some special stimulant to the nervous centres has seemed to be required. Thus we find that phosphorus has been administered in cases attended with great prostration of the vital powers, as in the latter stages of typhus fever, also in such chronic diseases of the nervous system as epilepsy, paralysis, melancholia, amaurosis, etc., occurring in debilitated subjects; and there is good evidence to show that in many of these nervous affections, the effect of phosphorus, properly administered, has been beneficial. * *

"The well-known fact that in cases where an unusual degree of wear and tear of the nervous system is being sustained, it is common to find an excess of phosphatic matter excreted in the urine, while the individual becomes increasingly weak, nervous, and irritable, appears to show that exhaustion of nervous force is in some way connected with a rapid oxidation and excretion of phosphorus from the system.

"Considering these points, we can see that there is reason in seeking to administer phosphorus, as an internal medicine, where we have reason to suspect that the nutrition of nervous matter may be failing, from a loss of its right proportion of this very essential ingredient.

"We give phosphorus for its restorative action over weak nerves, just as we give iron to nourish and restore blood that is weak and poor from lack of this constituent. * *

"For medicinal use, there are solutions of phosphorus in ether and also in almond oil. * *

"Another and very useful preparation of phosphorus is a pill, made by melting finely-divided phosphorus with fat and then covering the pill with an impermeable coating.

"Pills that I have seen and used, made by Messrs. Savory & Moore, contain 1-40th of a grain of phosphorus in each pill. Both Dr. Radcliffe and Dr. Althaus speak favorably of the good effect of phosphorus, given thus in very small doses, as a valuable tonic in many chronic nervous maladies. * *

"M. Tavignot, in France, has been in the habit of using phosphorus in the form of a pill, containing 1-70th of a grain, as a remedy in nervous, chlorotic, and strumous affections. In some neurotic and paralytic affections of the muscles of the eyeball and of the lachrymal nerve, M. Tavignot has used liniments and phosphorated oil with advantage; and, dropped into the eye, this oil is asserted, after some months' use, to have a solvent action on cataract. * *

"As a gradual tonic and restorer of failing nerve force, I prefer the hypophosphite of soda, or of lime, to the potash salt; and either of these salts appears to me to answer all the purposes of pure phosphorus, as an internal remedy, while, at the same time, they are more manageable and agreeable medicines. In cases of nervous depression and torpor, with at times shooting neuralgic pains, or, in other cases, numbness and deadness of the limbs, as from feeble circulation, the hypophosphites prove useful, and the lime or soda salt can be given according to the way in which the stomach may seem to bear the one better than the other. When anæmia is present, the citrate of iron can be added to the hypophosphite of soda, or else the syrup of the hypophosphite of iron, or of iron with quinine, can be employed; and either of these syrups will prove an active tonic, removing neuralgic pains, chest oppression, and languor of circulation in a very evident way." * * —*Boston Med. and Surg. Journal.—St. Louis Medical Archives.*

---

TREATMENT OF EPILEPSY.—Dr. Brown-Séquard states (*Half-Yearly Abstract*) that his usual prescription for epilepsy is as follows:—

Iodide of potassium, one drachm; bromide of potassium, one ounce; bromide of ammonium, two drachms and a-half; bicarbonate of potash, two scruples; infusion of calumba, six fluid ounces. Mix. A teaspoonful of the mixture to be taken before each of the three meals, and three teaspoonfuls at bedtime, with a little water.

In syphilitic cases he increases the amount of iodide of potassium. In administering the bromides it is necessary to give a relatively larger dose at bedtime and smaller doses in the day, if sleepiness is caused. The medicine should be pushed till anæsthesia of the fauces is produced, and an acne, like eruption, appears on the face, neck, and shoulders, etc. The bromides should be continued for fifteen of sixteen months after the attacks have ceased. An occasional purgative ought to be given, and if any debility be produced by the use of the bromides, wine and nourishing food should be used, with cod-liver oil, arsenic, strychnia, etc., and the cold douche or shower-bath employed.—*St. Louis Med. Archives.*

---

THE efforts to establish a library for the American Medical Association, at Washington, we are happy to say, are successful; and the profession are requested to bear in mind that all works deposited there are in excellent keeping, and that the movement will be of great service in the establishing of the medical literature of America.—*Buffalo Med. and Surg. Jour.*

---

DR. J. A. ROSS reports, in the *Lancet*, a case of death in the Staffordshire Infirmary, from the administration of 20 drops of chloroform. The patient was a miner, 50 years of age, upon whom an operation was to be performed. The chloroform was pure, and was given by means of a cone lint. The pulse stopped suddenly, and the most persevering use of all appliances for restoring animation proved unavailing. The autopsy showed the heart to be rather large and flabby, but otherwise free from disease. The other organs were normal.—*Buffalo Med. and Surg. Journal.*

Effects of Copper on the System.—In a paper read before the Clinical Society of London, Dr. Clapton gave some interesting information concerning the effects of copper upon the system of workers in that metal. The presence of "distinctly marked green stains on the teeth, close to the gums, bluish-green perspiration, hair of a greenish hue, in old workmen, and green discharge from old ulcers," were described and exemplified by the presentation of patients and specimens. These phenomena were shown to be probably the results of absorption, assimilation, and elimination of the metal. It is a singular fact that workers in copper not only are free from any specific poisoning beyond a tendency to muscular debility, but that they escape cholera and choleraic diarrhœa, even in localities where those diseases are epidemic.—*N. Y. Medical Gazette.*

---

Excessive Opium Eating.—Dr. A. T. Schertzer, of Baltimore, Md., relates the case of a lady, 28 years of age, who had consumed in two years, *five thousand eight hundred and forty ounces* of laudanum, and spent *eleven hundred and sixty dollars* in purchasing it. Without pretending to hold her attending physician to an unjust responsibility, the question naturally occurs, as to whether or not this heroic consumption and lavish expenditure might not have been prevented by the employment of judicious means, in the first instance? Medical men should always bear in mind that this dangerous habit is easily acquired, and should guard, as far as possible, against it.

"While attached to the Medical Corps of the Navy," says Dr. S., "I have frequently met with natives of the East Indies who consumed opium with a voracity equal to that of the lady in question, but have never known a similar instance in this country."—*Medical and Surgical Reporter.*

